# Enhancing the Mechanical Properties and Aging Resistance of 3D-Printed Polyurethane through Polydopamine and Graphene Coating

**DOI:** 10.3390/polym15183744

**Published:** 2023-09-13

**Authors:** Chien-Chiang Tung, Yen-Hong Lin, Yi-Wen Chen, Fu-Ming Wang

**Affiliations:** 1 Graduate Institute of Applied Science and Technology, National Taiwan University of Science and Technology, Taipei 10607, Taiwan; 2 x-Dimension Center for Medical Research and Translation, China Medical University Hospital, Taichung 404332, Taiwan; roger.lin0204@gmail.com; 3 Graduate Institute of Biomedical Sciences, China Medical University, Taichung 406040, Taiwan; 4 Department of Bioinformatics and Medical Engineering, Asia University, Taichung 41354, Taiwan

**Keywords:** polyurethane, polydopamine, graphene, coating, aging resistance

## Abstract

Three-dimensional (3D) printing is a versatile manufacturing method widely used in various industries due to its design flexibility, rapid production, and mechanical strength. Polyurethane (PU) is a biopolymer frequently employed in 3D printing applications, but its susceptibility to UV degradation limits its durability. To address this issue, various additives, including graphene, have been explored to enhance PU properties. Graphene, a two-dimensional carbon material, possesses remarkable mechanical and electrical properties, but challenges arise in its dispersion within the polymer matrix. Surface modification techniques, like polydopamine (PDA) coating, have been introduced to improve graphene’s compatibility with polymers. This study presents a method of 3D printing PU scaffolds coated with PDA and graphene for enhanced UV stability. The scaffolds were characterized through X-ray diffraction, Fourier-transform infrared spectroscopy, mechanical testing, scanning electron microscopy, and UV durability tests. Results showed successful PDA coating, graphene deposition, and improved mechanical properties. The PDA–graphene-modified scaffolds exhibited greater UV resistance over time, attributed to synergistic effects between PDA and graphene. These findings highlight the potential of combining PDA and graphene to enhance the stability and mechanical performance of 3D-printed PU scaffolds.

## 1. Introduction

Three-dimensional (3D) printing is often used for industrial applications due to its flexibility and capability to provide ample mechanical strength and support after fabrication [1]. Furthermore, 3D printing is cost- and time-efficient and can provide design versatility [2,3,4]. It can fabricate scaffolds with lattice-like structures, providing weight reduction and ample mechanical strength and stiffness for numerous engineering applications such as automobile, aerospace, and biomedical engineering [5,6,7]. Polyurethane (PU) is an important biopolymer widely used in 3D printing applications, especially for coating or as a composite and adhesive material [8,9]. The advantages of PU include low volatility, low solvent content, low odor, low toxicity, low volatile organic compound emissions, and excellent coating effects [10]. Despite this, the main limitation of PU lies in the fact that it is a biopolymer comprised of urea and ether bonds, thereby restricting its performance due to physical-chemical degradation [11]. The principal drawback of 3D-printed PU substrates is the poor mechanical behavior that the final material exhibits. According to studies, the mixture of reinforcements such as nanoparticles and filler was the most followed procedure for its enhancement [12]. In addition, ultraviolet light has been shown to degrade the carbamate bonds (C-NH), thus resulting in brittleness, high viscosity, and loss of mechanical properties of the polymer [13]. Therefore, the lifespan of PU is drastically limited for applications that have long-term exposure to UV light or other forms of environmental stimulants, and thus we need to explore efficient ways to preserve the mechanical stability of PU.

To overcome the abovementioned problem, various additives have been introduced into PU to improve its performance [14]. Among them, carbon fiber, graphene, and silica have been shown to improve the wear resistance and tensile strength of PU [15]. Other additives, such as UV absorbers and light stabilizers, have been added, and results have shown that these additives could improve the stability of the carbamate bonds. Wei et al. pointed out that adding varying proportions of benzotriazole derivatives also improved the UV stability of PU [16]. Several studies have also reported that adding compounds such as amino acids, silanes, and organic bismuth can simultaneously improve the mechanical properties and UV stability of PU [17,18]. However, additive processing and fabrication are complex and have high production costs. In addition, adding many additives may affect the plasticity of PU, thereby limiting its full potential for applications.

Graphene is a single-layered, two-dimensional material formed by a network of carbon atoms [19]. It has excellent physical, chemical, and mechanical properties and has been widely used in multiple specialties, such as electronics, energy storage, and biomedical applications [20,21,22]. Graphene has been added to various biomaterials, and its role is to act as a reinforcing agent and an antioxidant, thus improving the mechanical properties and stability of the biomaterials [23,24]. However, graphene has high surface area ratios, strong π-π stacking, and matrix incompatibility, thus leading to graphene aggregation and restricting homogenous dispersion with the biomaterial. To solve this issue, scientists have tried to modify graphene to solve the aggregation issues [25]. Various modification approaches have been developed, such as the attachment of silane coupling agents and functional group grafting, but these methods are complicated and environmentally toxic [26]. To overcome these problems, novel environmentally friendly methods, such as hyper-dispersion and surface modification techniques, have been developed. The concept of surface modification is to introduce different functional groups onto graphene surfaces to enhance its compatibility with the polymer matrix, thereby improving its dispersion and performance. The advantages of surface modification include simplicity, cost-efficiency, and being environmentally friendly [27].

Polydopamine (PDA) has a similar chemical structure to melanin, which can act as a UV absorber to absorb UV rays and eliminate free radicals, thereby preventing the photodegradation of polymers [28]. A recent bioinspired modification idea processing derived from the adhesive strategy of mussels, in which molecules could be attached to surfaces of materials via a simple immersion method [29,30]. In addition, PDA contains abundant catechol units and amino functional groups, which can provide excellent dispersion and intense interaction with the surfaces of biopolymers [31,32]. Therefore, using PDA modification to improve polymer properties has attracted extensive attention recently. In the past, scientists often mixed PU with graphene to fabricate PU/graphene composites. However, Zhao et al. reported that PDA-functionalized graphene effectively improved the homogeneity and stability of PU dispersions, thus significantly enhancing the corrosive resistance of PU [33]. In addition, Zhang et al. found that PU/PDA combined with reduction and oxidation graphene nanocomposites exhibited excellent electrical conductivity, thermal stability, and mechanical properties and had better potential for engineering applications than previous methods [34]. Yang et al. prepared PU/PDA-coated graphene nanocomposites and demonstrated the importance of the PDA layer in improving PU composites’ tensile, thermal, electrical, and stability properties [35]. In addition, Chen et al. demonstrated that a multifunctional interfacial PDA layer led to simultaneous enhancement of stiffness, tensile properties, anti-static characteristics, and UV and thermo-oxidative resistance of nanocomposites at high temperatures [15].

**Figure 1:**
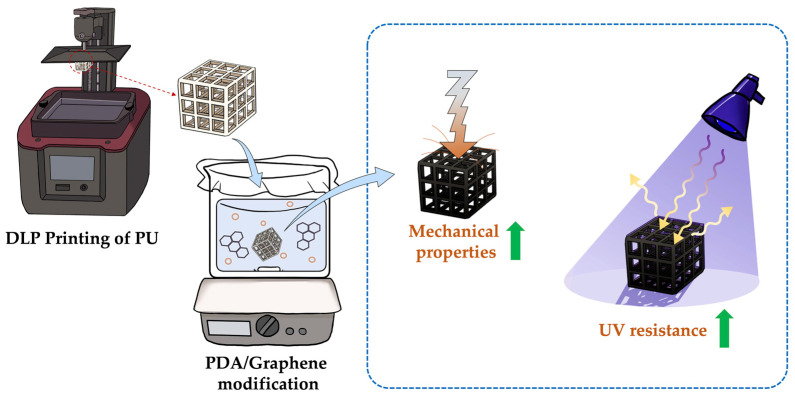
Schematic diagram of PU scaffold fabrication and PDA–graphene coating for enhancing mechanical properties and aging resistance behaviors.

In this study, we 3D-printed a robust composite material with excellent UV stability using PU with PDA/graphene coatings (Figure 1). Firstly, porous PU scaffolds were fabricated using stereolithography and then coated with PDA immersed in an alkaline solution. PDA molecules contain multiple functional groups, such as phenolic hydroxyl and amine molecules, which can interact with functional groups on the surface of the scaffolds via chemical bonding to achieve surface modification. The modified scaffolds were then homogenously coated with graphene, and the mechanical characteristics and UV stability were further evaluated and discussed in the following sections.

## 2. Materials and Methods

### 2.1. Fabrication and Surface Modification of 3D-Printed PU Scaffolds

Photocurable water-based PU resin (Alberdingk Boley, Krefeld, Germany) was first heated and dehydrated to 60% of its original weight. Then, 30% 2-hydroxylethyl methacrylate (HEMA; Sigma-Aldrich, St. Louis, MO, USA) and 1.5% 2,4,6-trimethylbenzoyl-diphenyl-phosphineoxide (TPO; Ciba Specialty Chemicals Inc., Basel, Switzerland) were mixed evenly, then added to the cooled PU and stirred. The above processes were carried out in a dark environment at room temperature. For the fabrication of scaffolds, 60 mL of the PU was loaded into the photocurable printing machine (Elegoo Mars 3, Elegoo Inc., Shenzhen, China) and fabricated according to our pre-designed Solidworks model. The design of the scaffold used in this study was 5.0 mm× 5.0 mm× 6.5 mm with 1 mm pores and a thickness of 0.25 mm. The scaffolds were fabricated layer-by-layer (50 µm per layer) with a 15 s UV exposure per layer. For surface modifications, the scaffolds were placed in different concentrations (0, 1, 2, 4 mg/mL) of 3-hydroxytyramine hydrochloride (dopamine HCl, Sigma-Aldrich, St. Louis, MO, USA) with 25 mM tris buffer (pH 8.5) and stirred overnight. Then, the scaffolds were rinsed and dried in an oven. For those groups containing graphene, 0.5 mg/mL of graphene was added to the dopamine HCl solution.

### 2.2. Scaffold Characterization

X-ray diffraction (XRD; Bruker D8 SSS, Bruker Corporation, Karlsruhe, Germany) analysis was used to analyze the crystalline structure and characteristics of the scaffolds. A 12 mm diameter by 2 mm height scaffold was fabricated for this study. Prior to XRD analysis, the scaffolds were first fixated onto the platform to ensure the accuracy and repeatability of the results. Copper Kα radiation (Cu Kα radiation) (λ = 1.5406 Å) was used as the source with a pre-determined operating voltage and current as per our previous protocols. The diffraction signals for this test were conducted in the range of 2θ angles (10–60°), and the corresponding diffraction intensities were then recorded and analyzed to determine the crystalline structure, orientation, and characteristics. Fourier transform infrared spectroscopy (FTIR; Nicolet 6700 FTIR, Thermo Fisher, Waltham, MA, USA) at a resolution of 4 cm^−1^ and range of 500–4000 cm^−1^ was used to analyze the molecular structures of the PU. To improve the signal-to-noise ratio, 32 scans were performed for each spectrum to ensure the reliability of the data, which was performed thrice, with the averages recorded. Compression tests were used to evaluate the mechanical properties of our composite materials. Compression tests were performed using an electronic universal testing machine (EZ-Test; Shimadzu Corp., Kyoto, Japan) that is able to provide constant, consistent compressive strength and rates. The samples were first fixated onto the holder of the testing machine, and a compressive rate of 1 mm/s was applied to the samples. The compressive force and deformation rates were recorded simultaneously and shown as a stress–strain curve emphasizing the Young’s modulus and fracture properties. Each group was tested in sextuplicate. Field emission scanning electron microscopy (FE-SEM; JEOL JSM-7800F; JEOL Ltd., Tokyo, Japan) was used to observe the composite materials’ surface morphology and elemental analysis. The samples were first fixated onto a self-designed platform, and carbon glue was applied homogenously to the surfaces of the samples. The samples were then left to dry to remove excess water or solvent, and a fine layer of metal coating was then deposited onto the surfaces to improve conductivity further. FE-SEM at an accelerating voltage of 3 kV was used to observe surface morphology.

### 2.3. UV Durability Testing

To investigate the UV durability of composites, we performed accelerated durability tests in an in-built chamber (Cure box, Vericom, Gangwon-do, Republic of Korea) equipped with a UV irradiation wavelength of 365 nm and 9 W/m^2^. The samples were placed into the in-built chamber and exposed to UV light for different durations (1, 3, 7, and 14 days) at 30 °C. FTIR, mechanical properties, and microscopic imaging were conducted at each timepoint to evaluate its durability and stability.

### 2.4. Statistical Analysis

One-way analysis of variance (ANOVA) was used to analyze differences between groups. Significant deviations were assessed using Scheffe’s multiple comparison test. A *p*-value of <0.05 was considered significant.

## 3. Results and Discussion

### 3.1. Characterization of PDA-Modified PU Scaffolds

**Figure 2:**
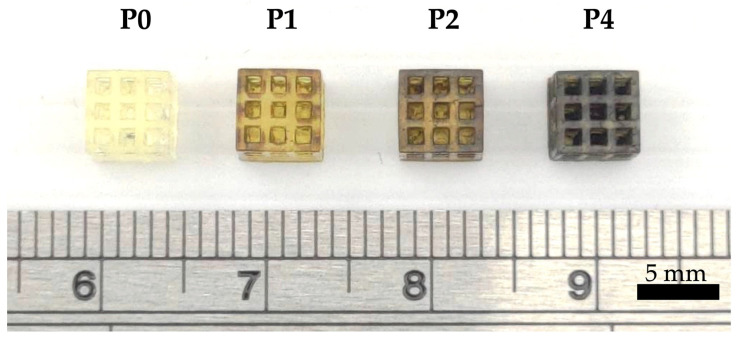
Three-dimensionally printed PU scaffolds with varying PDA concentrations. The scaffolds maintain their geometrical shape and porous structure while exhibiting changes in color according to PDA concentration. The scale bar is 5 mm.

Photo images of the 3D-printed PU scaffolds are shown in Figure 2. The scaffolds were designed to be a 5 mm× 5 mm× 5 mm cuboid with 1 mm× 1 mm× 1 mm pores and 0.5 mm struts. As seen in Figure 2, the struts and pores of the 3D-printed scaffolds were uniform, and the addition of PDA did not affect the structural characteristics of the scaffolds except for differences in physical appearances. Pure PU scaffolds (P0) were translucent and whitish, while adding PDA gave the scaffolds a dark, brackish appearance according to the concentrations of PDA added [36]. The observed color transition indicates successful surface modification. This color change is attributed to the deposition of PDA, a versatile polymer that can adhere to various substrates due to its adhesive and self-polymerization properties [37].

**Figure 3:**
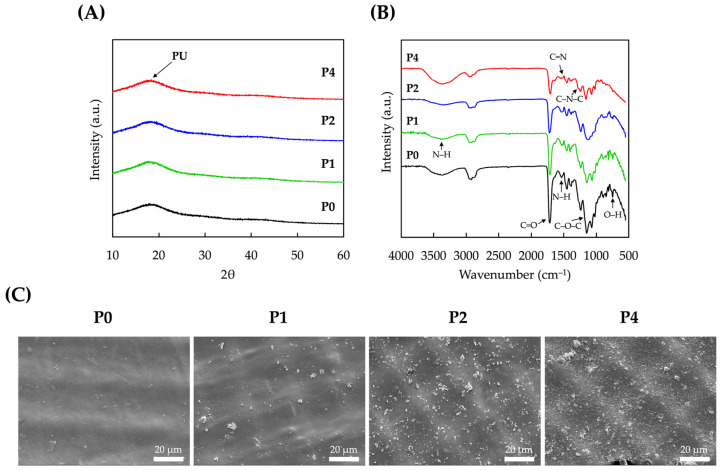
(**A**) XRD, (**B**) FTIR, and (**C**) SEM micrographs of pure PU (P0) and PDA-modified PU scaffolds (P1, P2, and P4). The scale bar is 20 µm.

The XRD results of PU and PDA-modified scaffolds are shown in Figure 3. P0 had a characteristic PU broad peak at 2θ = 18°, which was consistent with reports made by others stating that PU exists as a semi-crystalline polymer with an amorphous behavior [38]. For the rest of the groups, a characteristic broad peak at 18° was noted with no formation of other new peaks, thus indicating that the addition of PDA did not affect the crystallization behavior of our PU samples. FTIR was also used to analyze the surface chemistry of the samples. As seen in Figure 3B, the broad absorption bands at approximately 3300 cm^−1^, 1714 cm^−1^, and 1534 cm^−1^ corresponded to the N–H, C=O, and N–H bands of PDA, respectively. The bands at 1142 cm^−1^ and 748 cm^−1^ corresponded to the C–O–C and –OH bands of PU, respectively [39]. Furthermore, C=N and C–N–C were noted in the 1530 cm^−1^ and 1282 cm^−1^ ranges, indicating that PDA was successfully coated onto PU [40]. Taken together, it could also be noted that there were increasing intensities of PDA bands according to the concentrations of PDA noted, thus further indicating that different concentrations of PDA could be coated onto PU. Figure 3C shows the surface structure of PU and PDA-coated PU scaffolds. The SEM images showed that the surfaces had an undulated yet organized surface, which could be attributed to the fabrication process with UV light projection. It could be seen that PDA was deposited as aggregates of various sizes onto the surfaces of the scaffolds. As expected, increasing concentrations of PDA depositions showed increasing quantities of PDA deposition onto the surfaces of the scaffolds, giving the surfaces a rough appearance. Savchak et al. reported on the formation of PDA aggregations and their relationship with dopamine concentrations [41]. Interestingly, they noted that small PDA oligomers were noted at low PDA concentrations. These oligomers were then observed to act as seeds for the formation of larger PDA aggregates, and the diameter of PDA aggregates was also noted to increase exponentially with increasing PDA concentrations.

### 3.2. Mechanical Properties of PDA-Modified PU Scaffolds

**Figure 4:**
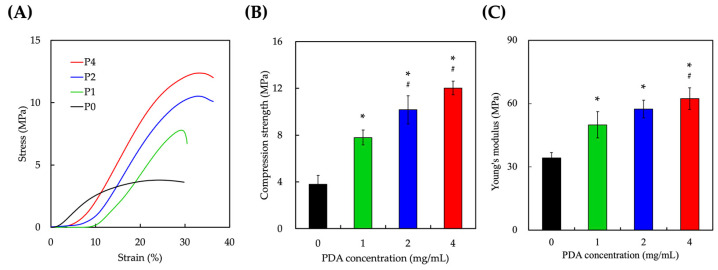
(**A**) Stress–strain curves of PU scaffolds with increasing PDA concentrations (P0, P1, P2, P4). (**B**) Compressive strength, and (**C**) Young’s modulus comparison of different scaffolds. * indicates a significant difference (*p* < 0.05) from 0% PDA. # indicates a significant difference (*p* < 0.05) from 1% PDA. Each group was tested in sextuplicate (*n* = 6).

The mechanical properties of the scaffolds were evaluated, and the stress–strain curves were as shown in Figure 4. As seen, P0 was able to support and sustain up to 3 MPa with 30% strain before scaffold failure. It was important to note that the stress–strain curves of P0 differ from the rest of the scaffolds. On the other hand, P1, P2, and P4 exhibited typical stress–strain curves of porous materials, with elasticity noted in the early stages of compression, represented by the gradual slopes, after which a plateau phase was noted before scaffold failure. As compared to P0, there was no elasticity phase noted, which is typical of brittle materials. Furthermore, Figure 4B,C show that increasing PDA concentrations enhanced compressive strength and Young’s modules exponentially. The compressive strength and Young’s modulus of P2 were 2.77 times and 1.53 times higher than P0, while P4 had 3.27 times and 1.87 times higher mechanical properties than P0. In fact, the PDA-coated layer could be filled into the pores of the construct to some extent by a large number of hydrogen bonds to grasp PU. This could further regulate a chemical reaction and increase the binding force between PU and PDA [42]. In addition, the PU scaffold coating processing was immersed in an alkaline environment, which is beneficial to the polymerization of PDA and can promote the polymerization reaction of PU and improve its strength [43]. The mechanical properties of a material affect how it behaves under mechanical stress. A material’s elasticity affects how it reacts under compressive stress, and its mechanical strength determines how much stress it can withstand before failing. The ductility of a material also plays an essential role in determining when it breaks when loaded beyond its elastic limit. Industrial applications heavily rely on materials’ mechanical performance for structural integrity. Elastic modulus influences how materials deform under load, impacting their ability to withstand stress. The enhanced strength of the PDA-modified materials is promising, as it broadens the range of applications they can effectively serve.

### 3.3. Effect of PDA–Graphene Modification

**Figure 5:**
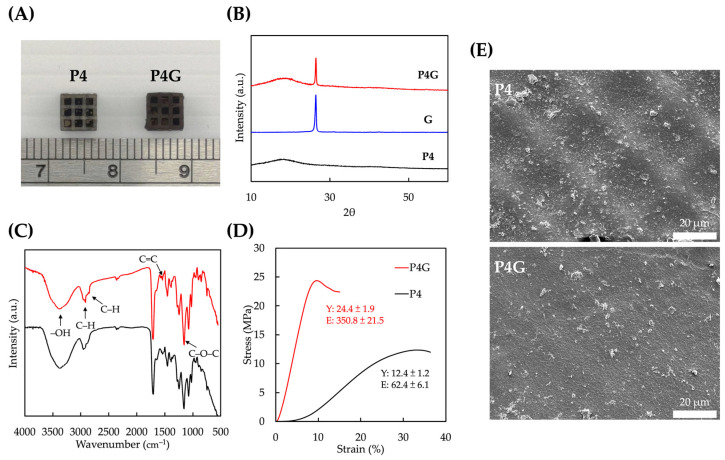
Synergistic effect of PDA–graphene modification. (**A**) Photograph of PU scaffolds before and after PDA–graphene coating. (**B**) XRD, (**C**) FTIR, (**D**) stress–strain curves, and (**E**) SEM micrographs of PU scaffolds before and after PDA–graphene coating.

Photo images of the scaffolds after PDA–graphene coating are shown in Figure 5A. PDA gave the scaffolds a dark, brackish appearance due to the self-polymerization characteristics of PDA as a poly-phenolic compound. Furthermore, graphene is composed of a single layer of carbon, thus making it highly light-absorbent. It absorbs both visible and infrared spectra, thus giving PU scaffolds a darker appearance. XRD spectra were used to characterize the physical characteristics of our composite scaffolds. Figure 5B shows a peak at 2θ = 25.6° for graphene and PU coating materials, with a d-spacing of 3.46 Å, corresponding to the presence of graphene in the composite material. This also implied that the graphene was coated on PU surfaces in a layered manner [44]. FTIR confirmed the successful coating of graphene on the surface of PU scaffolds by PDA. As shown in Figure 5C, the absorption peak at 3369 cm^−1^ could be attributed to the –OH groups on graphene. Furthermore, 1539 cm^−1^ also corresponded to the C=C and C-O-C bonds of graphene, thus further confirming the presence of graphene on PU surfaces [45]. The stress–strain curve from Figure 5D showed that adding graphene enhanced the scaffolds’ compressive strength and Young’s modulus. The ultimate compressive strengths before and after graphene treatments were 12.4 ± 1.2 MPa and 24.4 ± 1.9 MPa, respectively, and Young’s modulus was 62.4 ± 6.1 MPa and 350.8 ± 21.5 MPa, respectively. Graphene has been widely used in biomaterials due to its excellent mechanical properties, such as high yield strength and modulus [46]. It has been reported that graphene is an effective filler that can increase the material’s mechanical properties, including mechanical strength [47]. Furthermore, PDA was reported to be a good adhesive agent for surface modifications by increasing bonding interfaces for PU and graphene molecules. This concept stemmed from observing mussels’ strong attachment to walls. Our study showed that combining multiple materials can provide composite effects and expand the effects of biomaterials. The combination of PDA and graphene had a synergistic effect on increasing the mechanical properties of our scaffolds. From an industrial standpoint, the visually noticeable dark appearance of PDA–graphene-coated scaffolds can have implications across various sectors. In sectors like automotive and aviation manufacturing, where weight reduction and material efficiency are critical, the ability to create lightweight yet strong structures is of paramount importance. The use of graphene, with its high strength-to-weight ratio, has been investigated for potential applications in these industries [48]. In addition, SEM images showed that even without the presence of graphene (P4), PDA could self-polymerize on the surface of PU by forming nanoparticle-like structures and appearing as granules in the SEM images (Figure 5E). Due to the strong adhesion of PDA to graphene, the graphene nanosheets remained stably attached even under vigorous mechanical stress [49]. In addition, the PDA/graphene-coated layer can be applied for liquid/liquid separation, dye removal, and anti-corrosion [50].

### 3.4. Aging Resistance Effects of the PDA–Graphene Modified PU Scaffold

**Figure 6:**
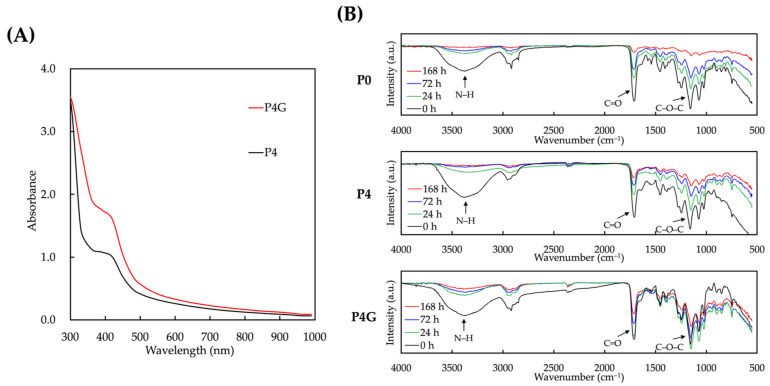
(**A**) UV absorption spectra of PU scaffolds before and after PDA–graphene coating. (**B**) FTIR analysis of scaffolds under UV exposure. PDA–graphene coating (P4G) offers protective effects against light-induced molecular structural changes.

The UV absorption capabilities of the scaffolds were evaluated at the wavelength range of 300–1000 nm, as shown in Figure 6A. As seen, the absorption value of P4G was relatively higher than that of P4. Graphene has excellent light absorption capabilities, especially in the visible and near-infrared ranges, due to its π-π* electronic transitions and surface plasmon resonance. Furthermore, PDA and graphene may form a synergistic effect in the composite, further enhancing absorption capabilities [51]. The presence of graphene may have changed the arrangement or ionic structure of the PDA molecules, which could lead to increased light absorption [52]. Taken together, this absorption enhancement may help improve the material’s performance, especially in areas such as light absorption and energy conversion. Decaying tests were performed on the scaffolds under different irradiation conditions (24, 72, and 168 h), as shown in Figure 6B. FTIR results showed that the uncoated group P0 underwent the most changes, with FTIR peaks decreasing gradually over the entire irradiation duration. The PDA coating offered a certain degree of protection, as seen from the lower decay noted in the P4 groups. Furthermore, there were no significant changes in the peaks of P4G, thus indicating that PDA and graphene could provide synergistic protective effects against light- and temperature-induced molecular structural changes, including changes in functional groups.

**Figure 7:**
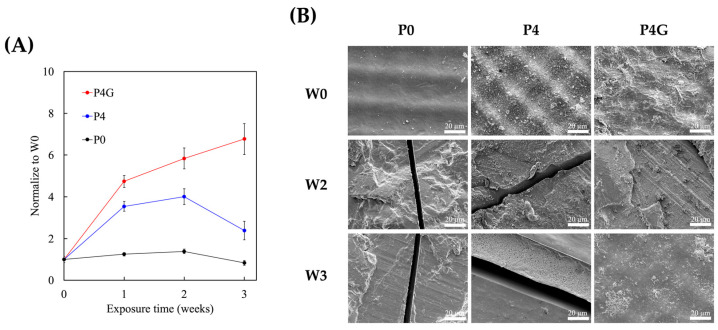
(**A**) Ultimate compressive strength changes in PU scaffolds under UV exposure over three weeks. P4G maintains increased mechanical strength even after prolonged irradiation. (**B**) SEM images of PU scaffold surfaces after UV exposure. P4G surfaces remain smooth without fracture fault lines, showcasing the strengthening effect of graphene in preventing crack propagation. The scale bar is 20 µm. Each group was tested in sextuplicate (*n* = 6).

The mechanical properties of the various scaffolds were evaluated after 1, 2, and 3 weeks of UV exposure, and the results are shown in Figure 7. P4G had the highest ultimate compressive strength at week 1 of exposure. Both P4 and P4G had increased ultimate compressive strength during the week of exposure. However, in the second week, both P0 and P4 were noted to have stable and stagnant ultimate compressive strength, while P4G continued to have increased ultimate compressive strength. At week 3, both P4 and P0 were observed to have decreased ultimate compressive strength, while P4G continued to have increased mechanical strength. The materials used in this study were inherently photocurable, meaning they can polymerize or cross-link when exposed to light of a specific wavelength to form a solid structure. As the material continues to be irradiated, it may have undergone a process of adaptation and rearrangement, making its internal structure denser and more organized, leading to increased compressive strength. This rearrangement may also partially counteract the detrimental effects of decay, and by the second week, these responses may have reached equilibrium. However, in the third week, the effects of UV decay may have overcome the rearrangement capabilities of P0 and P4, thus causing decay to occur. For the P4G group, adding graphene provided a sustained strengthening effect to the material, maintaining its stability even during a long-term decaying process. We found that the PDA–graphene modification significantly enhanced the UV stability and mechanical properties of the 3D-printed PU scaffolds. Before UV exposure, the strain measurements were as follows: P0 at 30%, P4 at 33.2%, and P4G at just 10.1%. However, after 3 weeks of exposure, there was a notable reduction in these strains: P0 plummeted to 8.6%, P4 to 10.4%, and P4G slightly increased to 11.8%. The increased strain post-UV exposure in the P0 and P4 samples may cause surface degradation or cracking due to UV irradiation. This effect suggests that these samples became more brittle and less flexible after UV exposure. Conversely, despite UV exposure, the minimal change in strain for the P4G sample underscores the protective and reinforcing effects of graphene. This highlights that the PDA–graphene modification provides UV resistance and imparts greater mechanical resilience to the scaffold. In addition, SEM was used to observe post-irradiation surface morphology changes. As seen from Figure 7B, fracture fault lines were observed in P0 and P4 after 2 weeks of irradiation, while the surfaces of P4G were smooth, with no fracture fault lines observed after 3 weeks of irradiation. This was consistent with our results above, whereby graphene strengthened the mechanical properties of scaffolds. The 2D planar structure of graphene exhibited excellent mechanical properties, which can resist the expansion or propagation of cracks to a certain extent, thereby slowing down or preventing the formation of fracture fault lines [53]. This effect, known as “fracture arrest” or “fracture absorption,” is an essential concept in materials science and engineering. Graphene’s high mechanical strength and flexibility allow it to effectively disperse stress, thereby preventing further crack propagation and further improving its overall mechanical properties.

## 4. Conclusions

In this study, we successfully developed a method for enhancing the UV stability and mechanical properties of 3D-printed PU scaffolds by coating them with PDA and graphene. The PDA coating, achieved through surface modification, enabled effective bonding between PU and graphene, facilitating the deposition of graphene nanosheets onto the scaffold surfaces. The resulting composite exhibited significantly improved compressive strength and Young’s modulus compared to pure PU scaffolds. Furthermore, the PDA–graphene-modified scaffolds demonstrated enhanced UV durability, with their mechanical properties remaining stable even after prolonged UV exposure. This phenomenon was attributed to PDA’s UV-absorbing properties and graphene’s mechanical reinforcement capabilities. This study’s results underscore the potential synergistic effects between PDA and graphene in enhancing the performance of 3D-printed materials. The introduced coating method offers a cost-effective and environmentally friendly approach to improving polymer composites’ stability and mechanical strength. As 3D printing technology continues to evolve, these findings have implications for developing durable and high-performance materials for various applications, including automotive, aerospace, and biomedical engineering. Further research could delve into optimizing the PDA–graphene coating process and exploring its applicability in other polymer systems, paving the way for innovative advancements in material design and engineering.

## Data Availability

Data are available in a publicly accessible repository.
